# The Effects of Residency and Body Size on Contest Initiation and Outcome in the Territorial Dragon, *Ctenophorus decresii*

**DOI:** 10.1371/journal.pone.0047143

**Published:** 2012-10-15

**Authors:** Kate D. L. Umbers, Louise Osborne, J. Scott Keogh

**Affiliations:** Research School of Biology, Australian National University, Canberra, Australian Capital Territory, Australia; CNRS, Université de Bourgogne, France

## Abstract

Empirical studies of the determinants of contests have been attempting to unravel the complexity of animal contest behaviour for decades. This complexity requires that experiments incorporate multiple determinants into studies to tease apart their relative effects. In this study we examined the complex contest behaviour of the tawny dragon (*Ctenophorus decresii*), a territorial agamid lizard, with the specific aim of defining the factors that determine contest outcome. We manipulated the relative size and residency status of lizards in contests to weight their importance in determining contest outcome. We found that size, residency and initiating a fight were all important in determining outcomes of fights. We also tested whether residency or size was important in predicting the status of lizard that initiated a fight. We found that residency was the most important factor in predicting fight initiation. We discuss the effects of size and residency status in context of previous studies on contests in tawny dragons and other animals. Our study provides manipulative behavioural data in support of the overriding effects of residency on initiation fights and winning them.

## Introduction

Identifying factors that influence the outcomes of costly contests is a classical field in evolutionary biology [1]–[3]. Asymmetries between males in body size and residency status are associated with an individual's likelihood of winning fights and as such are deemed determinants of contest success [4]. Size differences between opponents predict contest outcome in many species [5]–[7]. In sand gobies for example, males fight over nest sites and winners are on average 16.3% larger than losers [8]–[10]. However, other factors can override the effects of body size, such as prior contest experience [11] or being a territory holder (resident) [6], [12]–[14]. Prior contest experience may provide individuals with the opportunity to learn about their own fighting ability through self-assessment and predict their likelihood of winning [11]. Residency may also strongly influence the outcome of contests where residents are more likely to win contests over non-residents. Several hypotheses explain why this is so: (1) better males are intrinsically more likely to be territory holders; (2) residency status is a conventional cue by which to settle contests (‘bourgeois strategy’) [15], [16]; (3) residency leads to changes in the intrinsic quality of the resident [14], [17]; and/or (4) residents place greater value in their own territory than opponents place in the resident's territory because of their experience with it [13], [18].

In lizards contests can be highly aggressive and costly and the factors that determine the outcomes of these interactions are predictable in many species [19]–[23]. For example, residency is an important determining factor in contests between male snow skinks where the majority of interactions between size matched males are won by the resident [13]. Also, in the common wall lizard (*Podarcis muralis*) residency and size are both important determinants of contest outcome where again, residents and large males are more often victorious [24]. The factors that determine the outcomes of contests may not be the same as those that determine whether or not a fight is initiated. In Augrabies flat lizards (*Platysaurus broadleyi*), for example, in the initiation stages of contests, ultraviolet colouration is paramount [25] but colouration coupled with large size determines the winners of contest [26].

To investigate the determinants of contest outcome and initiation in the tawny dragon, *Ctenophorus decresii* we focused on the importance of size and residency status and how these interact to determine contest outcome and initiation. Specifically, we tested the hypotheses that contest outcome and contest initiation are determined by one of the following factors: (1) the relative size of opponents and (2) residency status. These traits were selected because size and residency status vary between individuals in nature and given the literature on lizard contest could each contribute to an individual's chance of winning or propensity to initiate contests. We predicted that large males and residents would be more likely to initiate and win fights.

## Methods

### Study animal

*Ctenophorus decresii* is a small arid zone agamid from Southern Australia [27]. Males are highly territorial and aggressive towards other males. Fights between males involve threat postures such as the elevation of the body with the back arched, lateral compression, lowering of the gular region and erection of nuchal and vertebral crests. Dynamic components include hind-leg push-ups where the rear of the animal is lifted in the air with the tail coiled over the back. This often occurs in conjunction with head bobbing and forearm waving [20], [27], [28]. Contests often escalate to wrestling with males locking jaws, however, this does not regularly result in injury (Osborne pers. obs.). The order of these components is not set and any of them may be absent in a contest (Osborne pers. obs.).

We collected 24 adult male *C. decressii* from the Flinders Ranges in South Australia (snout-vent length: 72 mm to 89 mm, 80.76±3.84 mm) in 2000 and in 2001. As they were mature adults, these lizards are likely to have had experience in antagonistic interactions before capture. Lizards were caught by noosing using waxed dental floss on the end of a 5 m telescopic fishing pole, and immediately transferred to calico bags. All individuals were recognisable by their unique gular patterns and colouration, and so it was not necessary to mark them, though colour does not inherently signal aggression (Osborne et al unpublished data). Animals were housed individually in outdoor enclosures for the duration of spring and summer (September 2003 to March 2004) in Canberra, Australia and the intervening two years are not likely to have effected our outcome. The enclosures were 2 m in diameter and divided in half with one male in each side. The divider prevented males from visual or chemosensory contact with other males while in their home enclosures. Enclosures had a natural soil substrate, with tussock grass, refugia and basking sites provided in the form of rocks and roof tiles. Food and water were available *ad libitum* in their home enclosures. Wild insects were available as a food source, which supplemented their diet of captive-bred crickets. Experiments were conducted in January and February 2004 between 10 am and 2 pm when the animals were naturally active.

### Experimental design

Twenty-four males were sorted into six size-matched groups of four individuals according to a size index. This method of size matching was used as it incorporated variability of the different measures of body size. In addition snout-vent length, mass and head-width were included as they are all potentially important in determining male contest outcomes [8], [22]. The index was calculated by running a principal components analysis with data for mass, snout-vent length and head width, and ranking the regression factor scores obtained.

The first PCA accounted for 77.7% of the variation. The regression factor scores from the first PCA were highly correlated with all the direct measurements of size (snout-vent length: *r* = 0.868; mass: *r* = 0.884; head width: *r* = 0.891).

We manipulated two factors (size and residency status) in five treatments: resident versus size-matched non-resident (R = NR); non-resident versus size-matched non-resident (NR = NR); resident versus larger non-resident (R<NR); resident versus smaller non-resident (R>NR); and small non-resident versus larger non-resident (NR>NR). In total 60 contests were conducted, 12 in each treatment. Each lizard underwent each treatment once only and was paired with a different lizard each time. Therefore, no pair interacted more than once and no lizard performed the same task more than once. Four interactions were conducted a day with a two day rest period between treatments for each group.

To assign lizards to the three size-asymmetrical treatments, animals were first divided into six size-matched groups of four and then the three groups of smaller lizards were paired with the three groups of larger lizards so that the size difference was constant (i.e. the smallest group of lizards with the smallest of the three larger groups of lizards). This produced three groups of eight, each consisting of four size-matched small lizards and four size-matched large lizards. The mean differences in size index for each group were: 1) X±SE = 1.911±0.076, N = 12; 2) X±SE = 1.486±0.064, N = 12; 3) X±SE = 1.844±0.140, N = 12. The order of treatments was different for each group to account for any possible order effects. For the three groups the order of treatments was: Group 1) NR>NR, R<NR, R>NR; Group 2) R<NR, R>NR, NR>NR; Group 3) R>NR, NR>NR, R<NR. For the two size-matched treatments, animals were divided into six size-matched groups of four. The order of treatments was different for each group to balance any possible order effects. For three groups the order was: N = NR, R = NR, and for the other three the order was R = NR, NR = NR.

### Behavioural observations

Experimental interactions were conducted in the outdoor enclosures, and to maintain consistency, experiments were conducted on days of clear weather and by the same person (Osborne). Each animal's home enclosure was used to test the effects of residency, and other enclosures (enclosures with no resident male) were used to test the effects of size independent of residency. All observations were made from behind a screen using a Dictaphone. In all contests, handling effects were kept equal. Animals were caught in the morning and kept in calico bags in the shade to ensure body temperatures were equal and since experiments were conducted in the height of summer and in the hottest part of the day (Canberra February average 25°C (Bureau of Meteorology 2012), lizard body temperature was close to optimal (*C. decressii* can be found active at temperatures as low as 20°C, Osborne, pers. obs). Both contestants were placed in a separate compartment of a cardboard box then placed into the enclosure with the lid opened so that animals could exit when they were ready. The starting interaction time was taken from when both animals could see each other; this was when the first signs of excitement, such as raised crests, were seen.

Interactions were analysed from recordings noting the contest winner and initiator, and the duration of the contest. Contests were stopped once a winner was determined based on continuous assertive behaviour such as an alert or aggressive posture. Losers were recognised by their lack of aggressive posturing, for example lowered crests, and fleeing to refugia when the other lizard postured or approached. Number and diversity of aggressive behaviours were scored according to the following index: bite (3), hind-leg push-up (lowering of dewlap, lateral compression, slow push-ups, and tail coiling) (3), chase (2), aggressive posturing (raising of nuchal or vertebral crests, back arching, lateral compression, lowering dewlap) (2), jerky walk (2), and tail flick (1) [29]. These scores are a conservative index of aggression and analogous to standard scoring systems used in other studies of lizard contests [30], [31]. Although more than one display may have been performed per interaction, individual display bouts were distinct, with animals returning to normal posture afterwards. Bouts of combat were also distinct with animals retreating to rest and bask between bouts. Most contests were resolved without physical fighting, biting occurred infrequently and involved a short nip to the base of the tail, but no scale damage or other physical trauma resulted.

### Statistical analyses

Statistical tests were conducted using R [32]. In R we generated several candidate generalised linear mixed models to determine which factors determine contest outcome. To account for repeated measures of lizards we included ‘individual’ in the model as a random factor. The response variable was ‘won or lost’ (winner was based on which lizard had the larger aggression score) and we included the two factors we manipulated (size (larger or smaller) and residency (resident or non-resident)) in the model. We also included which lizard initiated the fight (initiator, not initiator) in the model. It was not always possible to tell which animal initiated a contest so some data were missing across treatments for that variable (NR = NR: *N* = 11; R = NR: *N* = 10; NR>NR: *N* = 12; R<NR, *N* = 10; R>NR, *N* = 9) but the differences are so slight that we do not expect this to bias our results.

### Ethics statement

By necessity, contest length varied between bouts with a mean contest duration of approximately 12.6 minutes (range 7.6–19.0 minutes). All contests were stopped as soon as a clear winner was determined based on the behaviours outlined above. Animals could easily escape to a retreat site during the encounter, if desired. Sample sizes were kept to a minimum. All work was carried out as part of this project was done under the approval of the Australian National University Animal Experimentation Ethics Committee (F.BTZ.37.01) and with research permits from Environment ACT (permit number LT1999008). Collection of animals was conducted within the guidelines of South Australia National Parks and Wildlife Service under permit M24494. Animals were housed under the guidelines of Environment ACT (Australian Capital Territory) under permit K8164. The experiment complies with all current laws in Australia and approval was successfully sought from all necessary sources.

## Results

**Figure 1:**
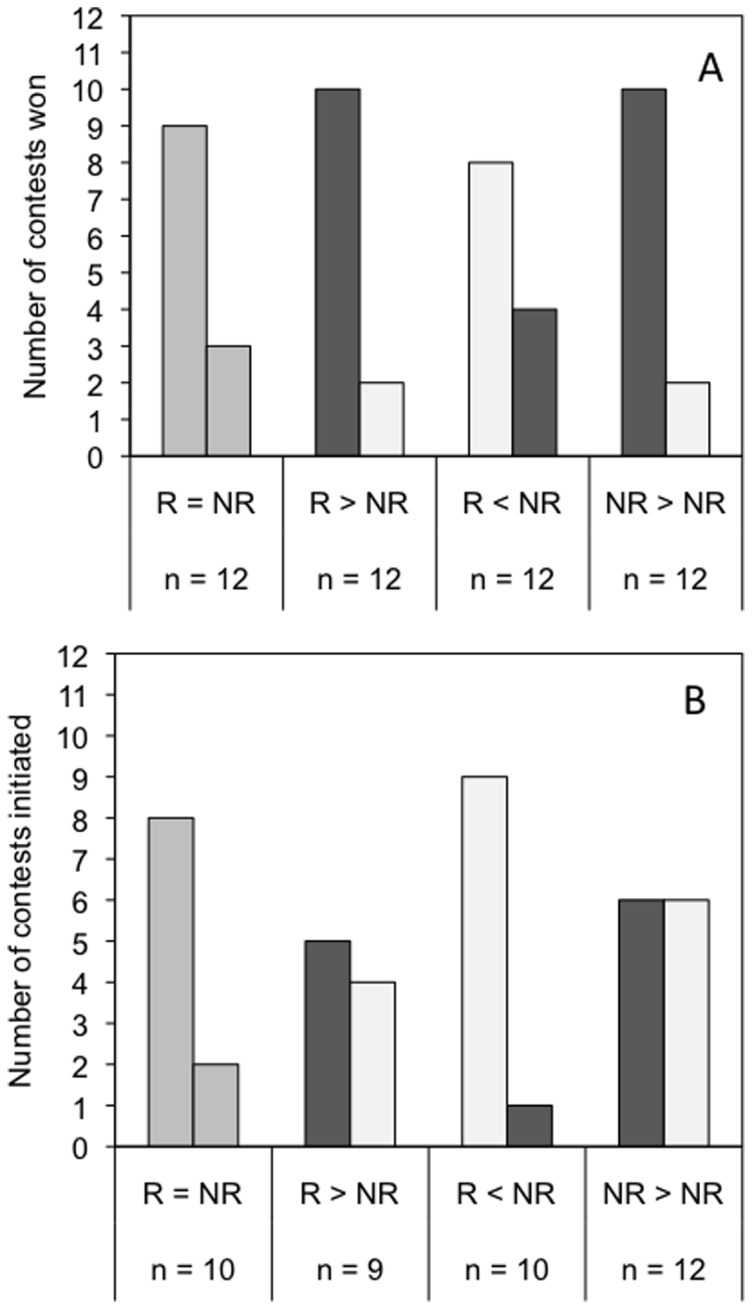
Number of contests won (a) and initiated (b) by the resident or the larger lizard across treatments. Dark grey columns = larger lizard; open columns = smaller lizard, light grey columns = lizards were size matched.

Overall, our analyses suggest that several factors we measured determine the outcomes of fights. We ran a model with each of the effects separately including individual as a random effect (Table 1). We ran models with interactions between factors to check that residence and body size affected contest outcome independently and found no significant interactions and thus excluded interactions from the final models presented here. Of our final two best models, one model included all factors (size, residency, initiation and individual as a random factor) and the other model included all factors except initiate (initiate had the lowest effect size in previous model) (Table 1). We compared AIC and AICc (derived from AIC) to choose the best models. AICc values are AIC values corrected for a sample size:factor ratio lower than 40 and rely on the number of effects (fixed and random), the number of observations and the AIC [33]. To calculate AICc we used the number of observations rather than the number of lizards because the repeated measures design was controlled for in the model by the inclusion of individual lizard as a random effect. The best model based on AIC was clear (20 AIC points clear of the nearest model) and included all variables (Table 1). Our model showed that body size had the largest effect size (β: 2.88±0.93, z = 3.09, p<0.001), followed by residency (β: 2.07±0.79, z = 2.62, p<0.01), and then by fight initiator (β: 2.06±0.87, z = 2.36, p = 0.02) (Figure 1a).

**Table 1:** Candidate models for determining the outcome of contests including: parameters measured in the model, Akiake's Information Criterion (AIC), the change in AIC compared to the best model, the −Log Likelihood of the model, AIC corrected for sample size to parameter (N:K) ratio of less than 40, change in AICc compared to the best model.

parameters included in model	AIC	AIC Δ	−Log Likelihood	AICc	AICc Δ
size+residency+initiate+individual as a random factor	69.01	0	−29.5	69.71175439	0
size+residency+individual as a random factor	89.02	20.01	−40.51	89.37294118	19.66118679
size+individual as a random factor	97.66	28.65	−45.83	98.01294118	31.59566845
initiate+individual as a random factor	126.8	57.79	−60.41	127.0424242	60.62515152
residency+individual as a random factor	157.1	88.09	−75.57	157.3068966	90.88962382
intercept+individual as a random factor (null model)	169.9	100.89	−82.93	170.1068966	103.6896238

We also looked at factors that contribute to the likelihood of an individual initiating a fight. In the model we included size and residency. We ran two models, one with and one without an interaction between size and residency both of which were very similar (AIC = 86.42 (AICc = X), df = 6 −LogLikelihood = 37.20, AIC = 85.45 (AICc = X ), df = 5, −LogLikelihood = −37.73, ANOVA: χ^2^ = 1.03, df = 1, p = 0.31). The interaction between size and residency was not significant and as such we present the simplest model, just including size, residency and individual as a random effect. Residency had a significant effect on initiating a contest (β: 1.50±0.63, z = 2.39, p = 0.02) whereas size did not (β: −0.97±0.55, z = −1.76, p = 0.08) (Figure 1b).

## Discussion

Body size, residency status, and initiating a contest were all significant predictors of winning a contest. While resident lizards were more likely to win than non-resident lizards (Figure 1a), our model shows that body size also had a strong effect, as did initiating a contest. When neither lizard was a resident, the larger male won more often. Residents were more likely to initiate a fight than non-residents and size differences did not determine whether an individual was more likely to initiate. Moreover, initiating a fight had a strong effect on contest outcome, but residency status and size were stronger effects on contest outcome than whether or not a lizard was the contest initiator.

Smaller residents were more likely to win contests than large intruders (residents also won more often than non-residents when they were larger or the same size as the intruder) (Figure 1a). The effect of residency was not due to differences in body temperature or diet as these were controlled. Also, our experimental design also ruled out the possibility of the intrinsic superiority of resident males [34], [35]. Moreover, residency was not used as a conventional cue as in the ‘bourgeois’ strategy, as contests often escalated to wrestling [15], [36]. However, it is possible that a superior knowledge of the territory provided an advantage or that the perceived cost of losing was greater for resident males [13], [17], [37], [38]. The same pattern has been shown in snow skinks where in size matched contests residents won 72% of contests regardless of size [13]. The strength of residency effects are also clear in female iguanas where knowledge of their burrow is thought to be important in residents winning fights over non-residents [39].

When residency was equal, body size was an important predictor of contest outcome in *C. decresii* (Figure 1a). Body size is an important determinant of contest outcome in many species, where the larger animal is presumably able to overpower the smaller one [10]. For example in 92% of New Zealand jumping spider (*Euophrys parvula*) contests the larger competitor won. Similarly, in threespine sticklebacks (*Gasterosteus aculeatus*), males just 15% heavier than their rivals won contests more often [40]. A physical advantage in *C. decresii* may be conferred by greater mass and strength or larger males with a correspondingly larger head width may give them an advantage in contests involving wrestling and biting [41] as in eublepharid geckos and sand lizards (*Lacerta agilis*), for example [42], [43]. Instead of body size, contests may be determined by strength, endurance or motivation [44], [45].

Coupled with the effects of body size and residency status patterns of contest initiation proved an important determinant of contest in *C. decressii*. Resident lizards were more likely to initiate contests and initiators were more likely to win. In red jungle fowl, cocks that initiate antagonistic interactions are also highly likely to be the winners of interactions [47]. Ligon et al (1990) suggest that this pattern indicates a method of pre-fight assessment where by competitors gather information about each other prior to contest initiation. So although we can rule out the use of residency status as a ‘bourgeois’ strategy there may be inherent features of residence that make them formidable competitors. In a previous study Osborne (2005) shows that in tawny dragons (*C. decressii*) signals relating to aggression (i.e. black chest patch) are not correlated with body size or condition but nevertheless are a strong predictor of contest outcome [46]. Finally, in the absence of a residency asymmetry small lizards were as likely to initiate contests as large lizards (Figure 1b). Similar size-independent initiation occurs in the velvet swimming crab (*Necora puber*), in which the relationship between size and its fighting ability can be variable and being larger is not always advantageous [44].

In summary, body size, residency and contest initiation, all are important determinants of contest outcomes in *C. decresii*. Large animals are more likely to win contests compared to small animals when both are non-residents, but residents are often able to overcome a size disadvantage. Residents are more likely to initiate and win fights but the mechanism conferring a resident advantage is unclear. Lizards may value investment in their territory leading to the aggressive motivation of residents being greater than that of intruders or alternatively residents may be inherently better competitors. Further work should aim to determine whether certain features of territories give resident tawny dragons the competitive edge over non-residents.
